
JOURNAL INFORMATION
==============================
NLM Title Abbreviation: Biospektrum (Heidelb)
Journal ID: Biospektrum (Heidelb)
NLM Journal ID: 9615685

Biospektrum

ISSN: 0947-0867
EISSN: 1868-6249

ARTICLE INFORMATION
==============================
PMCID: PMC8233609
DOI: 10.1007/s12268-021-1606-4
Article ID: 1606
Article version: 1
Subjects: Karriere, Köpfe & Konzepte

Buchrezension zu: The Doctor Who Fooled the World

Seifert Roland seifert.roland@mh-hannover.de

grid.10423.34 0000 0000 9529 9877 Medizinische Hochschule, Hannover, Deutschland
Electronic publication date: 2021 Jun 26
Print publication date: 2021
Volume: 27
Issue: 4
First page: 453
Last page: 453
Copyright: © Der Autor 2021
License: Open Access: Dieser Artikel wird unter der Creative Commons Namensnennung 4.0 International Lizenz veröffentlicht, welche die Nutzung, Vervielfältigung, Bearbeitung, Verbreitung und Wiedergabe in jeglichem Medium und Format erlaubt, sofern Sie den/die ursprünglichen Autor(en) und die Quelle ordnungsgemäß nennen, einen Link zur Creative Commons Lizenz beifügen und angeben, ob Änderungen vorgenommen wurden. Die in diesem Artikel enthaltenen Bilder und sonstiges Drittmaterial unterliegen ebenfalls der genannten Creative Commons Lizenz, sofern sich aus der Abbildungslegende nichts anderes ergibt. Sofern das betreffende Material nicht unter der genannten Creative Commons Lizenz steht und die betreffende Handlung nicht nach gesetzlichen Vorschriften erlaubt ist, ist für die oben aufgeführten Weiterverwendungen des Materials die Einwilligung des jeweiligen Rechteinhabers einzuholen. Weitere Details zur Lizenz entnehmen Sie bitte der Lizenzinformation auf http://creativecommons.org/licenses/by/4.0/deed.de.
License URL: https://creativecommons.org/licenses/by/4.0/

issue-copyright-statement: © Springer-Verlag GmbH Deutschland, ein Teil von Springer Nature 2021

==============================

**Pillen vor die Säue**
**Warum Antibiotika in der Massentierhaltung unser Gesundheitssystem gefährden**
**Rupert Ebner und Eva Rosenkranz**

*249 S., oekom, 2021. SC, 20,00 €.*
*ISBN: 9783962382063*
*Auch als E-Book erhältlich*

■ Zunächst eine Klarstellung: Der Begriff „Antibiotika“ ist ein Misnomer. Der Begriff besagt im wörtlichen Sinne, dass diese Arzneistoffgruppe gegen das Leben insgesamt gerichtet ist. Aber das trifft glücklicherweise nicht zu. „Antibiotika“ wirken nur gegen Bakterien. „Antibiotika“ sind also korrekterweise antibakterielle Arzneistoffe. Dies ist keine sprachliche Detailversessenheit, sondern von fundamentaler wissenschaftlicher Bedeutung, da der Begriff „Antibiotika“ Ärzte, Tierärzte und Landwirte dazu bringt, diese Arzneistoffe gegen alle möglichen Erkrankungen einzusetzen, z. B. auch solche, die durch Viren oder Pilze verursacht werden. Das führt dann leider zu Resistenzen gegen die verfügbaren Arzneistoffe. Die Folge davon wiederum ist, dass es inzwischen viele bakterielle Krankheitserreger gibt, die gegen die meisten antibakteriellen Arzneistoffe resistent sind. Das Resultat sind schwerwiegende Krankenhausinfektionen mit dem berüchtigten „multiresistenten *Staphyloccocus aureus* (MRSA)“, auf dessen Konto viele vermeidbare Todesfälle gehen.

Diese wichtige Begriffsbestimmung beschreibt dann auch schon das Kernthema des rezensierten Buchs. Es geht darum, dass der unkritische Gebrauch von antibakteriellen Arzneistoffen in der Zucht von Geflügel, Schweinen und Rindern dazu führt, dass es zu immer weitreichenderen Resistenzen gegen antibakterielle Arzneistoffe kommt - mit gravierenden Konsequenzen auch für den Menschen.

Ausgehend von dem im Fokus stehenden „Antibiotika“-Problem skizzieren die Autoren sehr anschaulich und mit vielen Beispielen die alles andere als appetitanregende Tierzucht und Fleischproduktion in Deutschland. Die damit verbundenen Probleme wurden im Sommer 2020 schlaglichtartig durch den COVID-19-Ausbruch in dem Schlachtbetrieb Tönnies aufgezeigt, der paradigmatisch für eine fehlgeleitete Fleischproduktion steht.

Aufgelockert wird das Buch durch zahlreiche Textboxen und grau unterlegte Texte mit dem Titel „Blickwechsel“. Hilfreich beim Lesen, das auch „kreuz und quer“ erfolgen kann, sind prägnante Unterüberschriften sowie sehr gute Zitate bekannter Persönlichkeiten zum Buchthema. Abgerundet wird es durch ein umfangreiches Quellenverzeichnis. Der Preis ist angemessen. Das Buch eignet sich als Geschenk für jeden Leser, der an gesunder Ernährung und nachhaltiger Landwirtschaft interessiert ist. Das Einzige, was der Rezensent wirklich vermisst hat, ist ein abschließendes Kapitel, was jeder einzelne Mensch als Konsument und Staatsbürger konkret im täglichen Leben tun kann und sollte, um die Entwicklung von weiteren Resistenzen gegen antibakterielle Arzneistoffe zu verhindern. Aber es ist letztlich einfach: Ohne antibakterielle Arzneistoffe hergestelltes Fleisch gibt es eben auch nicht zum Dumpingpreis, sondern zu einem deutlich höheren Preis. ■

*Roland Seifert,*
*Medizinische Hochschule Hannover,*
*seifert.roland@mh-hannover.de*

Diese Rezension erscheint **Open Access**.*

* **Funding note:** Open Access funding enabled and organized by Projekt DEAL.
**Open Access:** Dieser Artikel wird unter der Creative Commons Namensnennung 4.0 International Lizenz veröffentlicht, welche die Nutzung, Vervielfältigung, Bearbeitung, Verbreitung und Wiedergabe in jeglichem Medium und Format erlaubt, sofern Sie den/die ursprünglichen Autor(en) und die Quelle ordnungsgemäß nennen, einen Link zur Creative Commons Lizenz beifügen und angeben, ob Änderungen vorgenommen wurden. Die in diesem Artikel enthaltenen Bilder und sonstiges Drittmaterial unterliegen ebenfalls der genannten Creative Commons Lizenz, sofern sich aus der Abbildungslegende nichts anderes ergibt. Sofern das betreffende Material nicht unter der genannten Creative Commons Lizenz steht und die betreffende Handlung nicht nach gesetzlichen Vorschriften erlaubt ist, ist für die oben aufgeführten Weiterverwendungen des Materials die Einwilligung des jeweiligen Rechteinhabers einzuholen. Weitere Details zur Lizenz entnehmen Sie bitte der Lizenzinformation auf http://creativecommons.org/licenses/by/4.0/deed.de.

Funding note: Open Access funding enabled and organized by Projekt DEAL.

